# Mechanical evaluation of newly developed mouthpiece using polyethylene terephthalate glycol for transoral robotic surgery

**DOI:** 10.1007/s11701-015-0539-7

**Published:** 2015-11-04

**Authors:** Kazunori Fujiwara, Takahiro Fukuhara, Koji Niimi, Takahiro Sato, Hideyuki Kataoka, Hiroya Kitano, Hiromi Takeuchi

**Affiliations:** Department of Otolaryngology, Head and Neck Surgery Faculty of Medicine Tottori University, 36-1, Nishimachi, Yonago, 683-8504 Japan; Mechanical and Material Research Facility, Mechanical Engineering Division, Tottori Institute of Industrial Technology, Yonago, Japan

**Keywords:** Load, da Vinci, TORS, Mouthpiece

## Abstract

Transoral robotic surgery (TORS), performed with the da Vinci surgical system (da Vinci), has been classified as a surgical approach for benign and malignant lesions of the oral cavity and laryngopharynx. It provides several unique advantages, which include a three-dimensional magnified view, ability to see and work around curves or angles, and the availability of two or three robotic arms. At present, however, the da Vinci surgical system does not provide haptic feedback. The potential risks specific to the transoral use of the da Vinci include tooth injury, mucosal laceration, ocular injury, and mandibular fracture. To prevent such intra-operative tooth injuries, we created a mouthpiece made of polyethylene terephthalate glycol (PETG) individually shaped for the patient’s teeth. We compared the safety and efficacy of the PETG mouthpiece with those of a conventional mouthpiece made of ethylene–vinyl acetate (EVA). To determine the difference in tooth injury resulting from the two types of mouthpiece, we constructed an experimental system to measure load and strain. We measured the dynamic load and the strain from the rod to the tooth using the PETG and EVA mouthpiece. The rod was pressed against the tooth model outfitted with two types of mouthpiece and the dynamic load was measured with a load cell and the strain with a strain gage. The maximum dynamic load was 1.29 ± 0.03 kgf for the PETG mouthpiece and 2.24 ± 0.05 kgf for the EVA mouthpiece. The load against the tooth was thus less for the EVA mouthpiece. The strain was −166.84 ± 3.94 and 48.24 ± 7.77 με, respectively, while the load direction was parallel to that of the tooth axis for the PETG mouthpiece and perpendicular to the tooth axis for the EVA mouthpiece. The PETG mouthpiece reduced the tooth load compared with the EVA mouthpiece and the load direction was in parallel to the tooth axis. The PETG mouthpiece thus enhances tooth safety for TORS.

## Introduction

Robotic surgery using the da Vinci surgical system (Intuitive Surgical Inc., Sunnyvale, CA, USA) has gained popularity as a therapeutic procedure in many surgical fields, especially urological and gastrointestinal [1]. While its applications for head and neck surgery are still in the developmental stage, several reports have advocated the effectiveness of robotic surgery for thyroid and transoral surgery [2–9].

Transoral robotic surgery (TORS) with da Vinci surgical system has been used for the removal of pharyngeal and laryngeal cancers with the objective of improving swallowing and other functional as well as esthetic outcomes without worsening survival [2–9]. Several studies have demonstrated that TORS may be an effective alternative to open surgery and chemoradiation for oropharyngeal tumors [2, 3] in terms of improved cosmetic results, shorter hospital stay and preservation of swallowing function. TORS allows for a wide and clear view of the surgical field and 3D visualization of structures, thus enabling access to the tumor via a relatively small approach.

At present, however, the da Vinci surgical system cannot provide haptic feedback. Specific to the transoral use of the da Vinci results are the potential risks of tooth injury, mucosal laceration, ocular injury, and mandibular fracture. The safety record of the da Vinci surgical system in its application to thoracoscopic and laparoscopic surgery establishes a solid background of overall device safety [10, 11], but for TORS, several studies have reported occurrences of intraoperative tooth injury [12]. However, little has been published regarding evaluation of the intraoperative safety of TORS and the utility of various mouthpieces for prevention of injury since surgeons may use several types of mouthpieces during transoral surgery, including TORS, to reduce the risk of intraoperative tooth injury. In addition, TORS needs a wide surgical field so that a thin and firm mouthpiece is needed. We focused on a mouthpiece developed by us and made of polyethylene terephthalate glycol (PETG) (Erkodur^®^), which is currently used in the dental field as the material for mouthpieces for orthodontics [13–15] and evaluated the effectiveness of this PETG mouthpiece.

The first aim of this study was to examine the material properties of PETG (Erkodur^®^) and ethylene–vinyl acetate (EVA) (Erkoflex^®^) used for conventional mouthpieces by means of assessment procedures such as measurement of friction coefficient, Martens’ hardness test, and Young’s modulus. The second aim was to determine whether the mouthpiece made of PETG is more effective for tooth damage prevention than the conventional mouthpiece made of EVA (Erkoflex^®^) by measuring load and strain.

## Materials and methods

### Materials

**Fig. 1:**
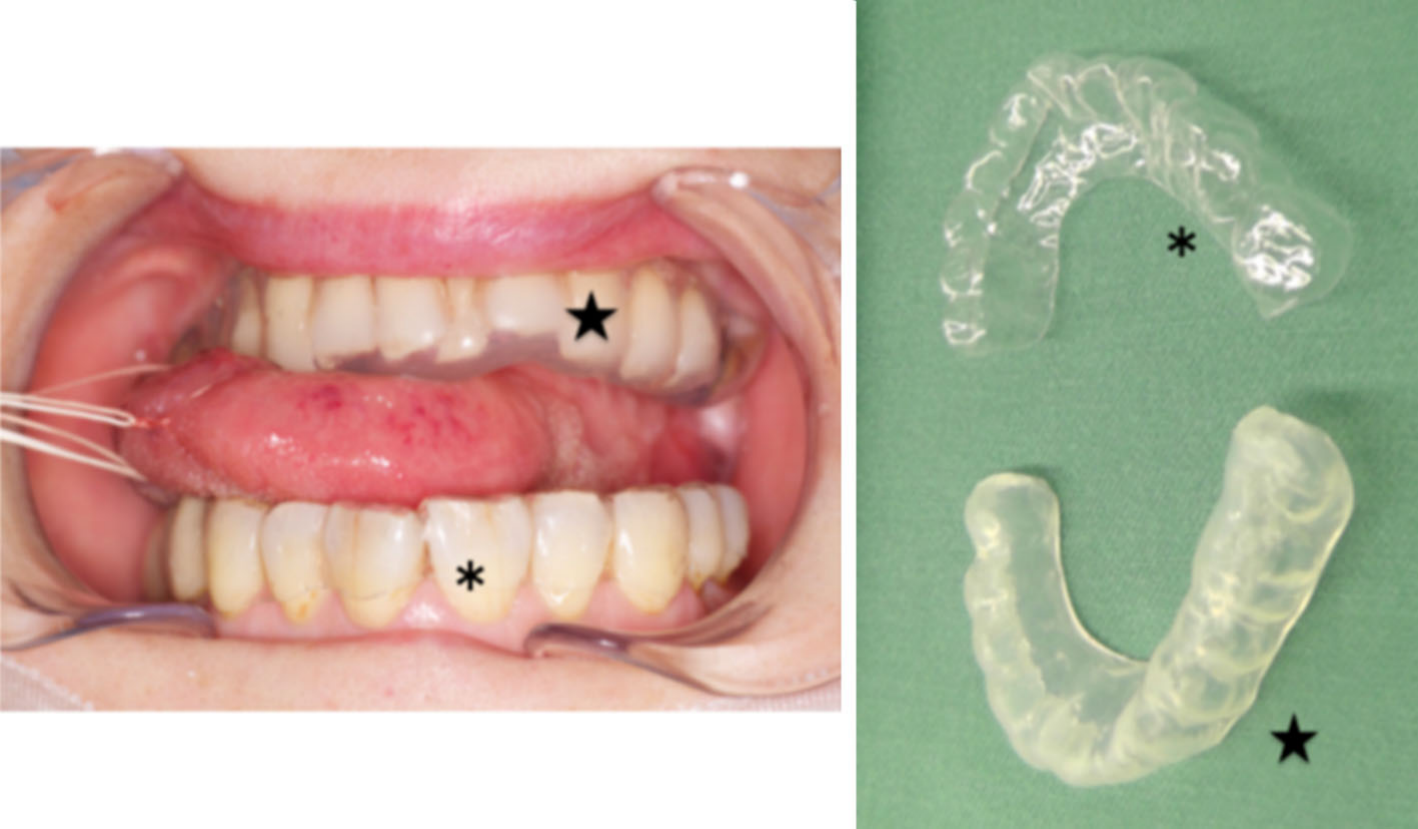
PTEG (Erkodur^®^) mouthpiece (*asterisk*) and EVA (Erkoflex^®^) mouthpiece (*star*)

Two thermoplastic materials, Erkodur^®^, 0.5 mm thick, and Erkoflex^®^, 3 mm thick (both from Erkodent Erich Kopp GmbH, Pfalzgrafenweiler, Germany), were selected as material for the mouthpieces used in this study (Fig. 1). Erkodur^®^ is made of PETG and Erkoflex^®^ is made of EVA.

### Material analysis

To measure the material properties of Erkodur (PETG) and Erkoflex (EVA), we used Martens’ hardness test (N/mm^2^) and Young’s modulus (N/mm^2^) in combination with a Nanoindentation Tester (ENT-1100a; Elionix Inc., Tokyo, Japan).

This measurement procedure involves the application of a prescribed load to an indenter in contact with a specimen. As the load is applied, the depth of penetration is measured. The depth of the impression and the known angle or radius of the indenter are used to determine the area of contact at full load. The hardness is then found by dividing the load by the area of contact, while the shape of the unloading curve provides a measurement of the elastic modulus.

### Measurement of friction coefficient

To clarify friction differences due to type of material and determine the effect of friction on load and strain, we measured the friction coefficients of Erkodur^®^ and Erkoflex^®^ using the Tribometer^®^ (Nanotec Corporation, Chiba, Japan) under the following conditions: velocity 1.00 cm/s; preload 5.00 N, temperature 21.1 °C; humidity 39.00 %; ball diameter 6 mm; ball material SUJ2 (chrome steel).

### Production of mouthpiece

The Erkopress ES 2002 (Erkodent Erich Kopp GmbH) was used to make the mouthpieces of PETG and EVA. The stone tooth model was placed in the device and filled with PETG and EVA with the spacer foil pointing towards the stone tooth model, for thermoforming and trimming. The resultant mouthpieces are shown in Fig. 1.

### Measurement of dynamic load

To understand as to what extent differences in the load on the tooth model depend on the mouthpiece material, we measured the dynamic load on the tooth. Dynamic load was defined as the force that is time dependent and quickly changes in magnitude or direction as determined with a load cell system is used. A load cell is a piezoelectric quartz crystal sensor used to measure force in terms of the piezoelectric effect. This effect refers to the linear electromechanical interaction between the mechanical and the electrical state in crystalline materials with no inversion symmetry. Piezoelectricity is the electric charge that accumulates in certain solid materials, such as crystal, in response to applied mechanical stress.

The Loadcell 9317B (Kistler Japan Co., Ltd., Tokyo, Japan) with the appropriate load amplifier (5019B130; Kistler Japan Co., Ltd.) was used for measuring dynamic load. The data acquisition recorder (Memory High Coder, Hioki Denki Co., Ltd., Tokyo) used for our study allows for direct and continuous recording with simultaneous graphical visualization of the dynamic load. The data of the dynamic load was recorded 1,000 times per second, previewed from the data recorder and stored on a personal computer.

**Fig. 2:**
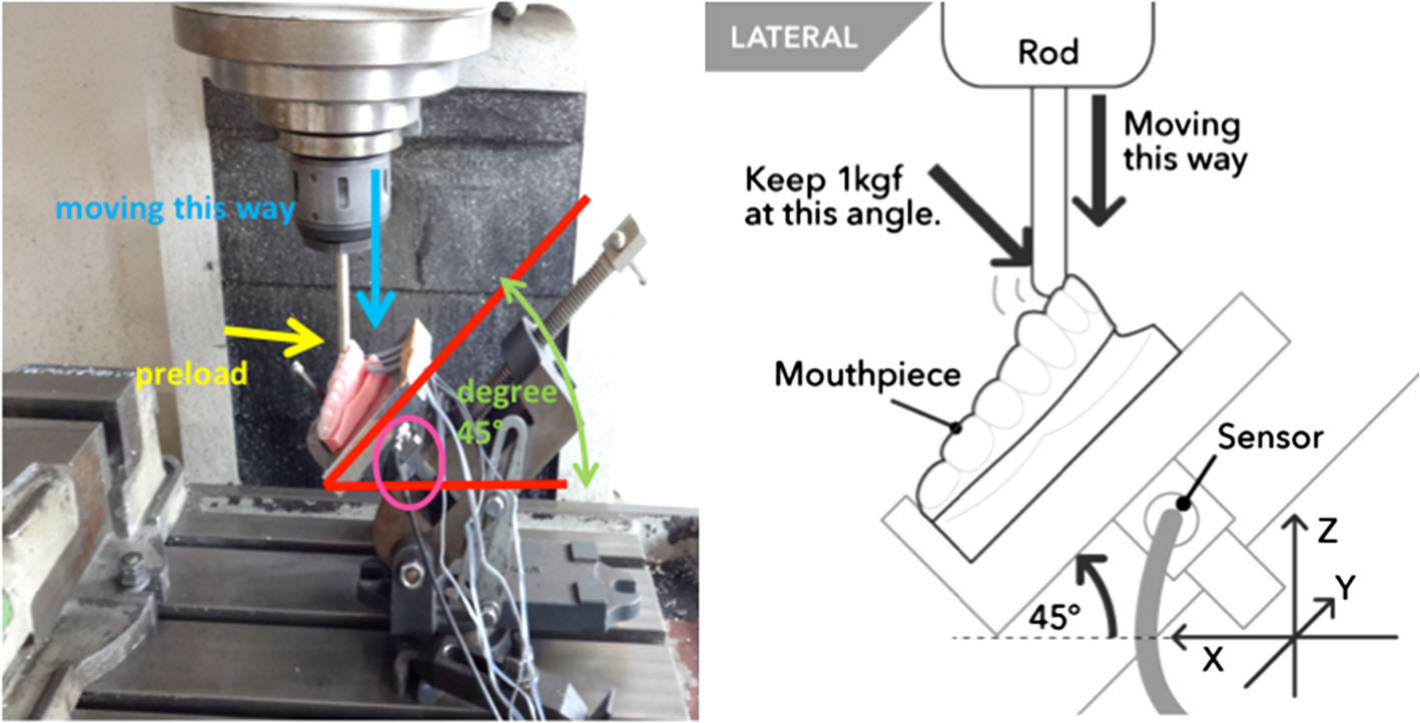
Measurement of load on tooth model

The stone tooth model with the mouthpiece was placed on the instrument table at an incline of 45°. A 12-mm metal rod was pressed against the tooth model outfitted with either of the two types of mouthpiece with a preload of 1 kgf and slid down at a rate of 200 mm/min. The Loadcell was placed under the instrument table and used to measure the dynamic load acting on the stone model. The measurements were performed at room temperature (25 °C) and repeated five times for each mouthpiece. The force was stabilized within 10 s and was set as the maximum force for purposes of this study (Fig. 2).

### Measurement of strain

To identify the differences in the strain exerted on the tooth according to the mouthpiece material, we measured the strain on the bilateral central incisor. Strain is defined as the change in length after application of stress to the initial unstressed reference length and is expressed as a ratio. A strain gage is the element that senses this change and converts it into an electrical signal reflecting changes in strain gauge resistance as it is stretched, or compressed, similar to such changes in a wire. When wire is stretched, its cross-sectional area decreases; therefore, its resistance increases.

A strain gauge (FLA-03-17-3LT; Tokyo Sokki Kenkyujo Co., Ltd., Tokyo, Japan) with the appropriate load amplifier (5019B130; Kistler Japan Co., Ltd.) was used for measuring strain. For direct and continuous recording with simultaneous graphical visualization of the dynamic load, a data acquisition recorder was used. The data were then displayed by a data recorder (8860-50; Memory High Coder, Hioki Denki Co., Ltd.) and stored on a personal computer. Since the endoscope and instruments quite often touch the incisors during TORS, the strain gages were applied to the bilateral four central incisors.

**Fig. 3:**
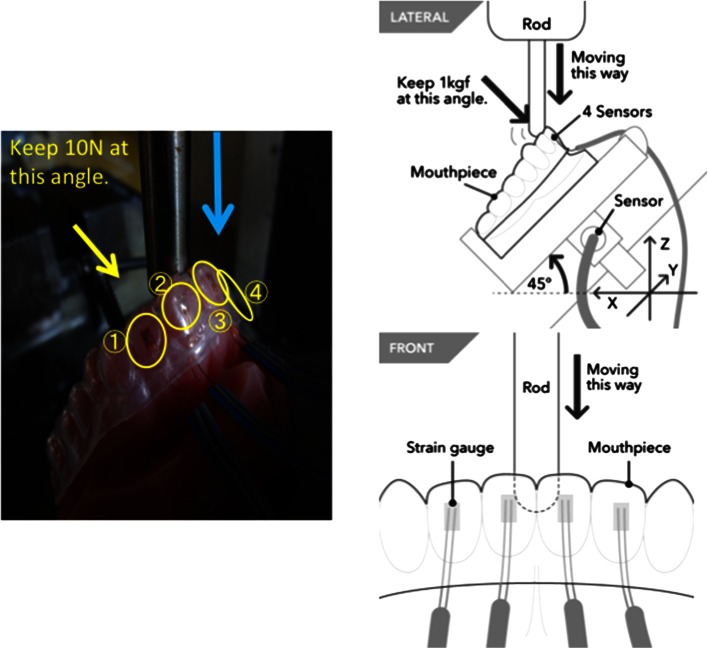
Measurement of strain on bilateral center incisors

The stone model with the mouthpiece was placed on the instrument table at an angle of 45°. The 12-mm metal rod was pressed against the tooth model outfitted with either of the two types of mouthpiece with a preload of 1 kgf and slid down at a rate of 200 mm/min. The strain was measured with the strain gauge (Fig. 3). The measurements were performed at room temperature (25 °C) and repeated five times for each mouthpiece. The force was stabilized within 10 s and was set as the maximum force for purposes of this study.

### Statistical analysis

All values are shown as the mean ± SD, and statistical analyses were performed using a standard *t* test with a significance level of 5 %.

## Results

### Material analysis

**Fig. 4:**
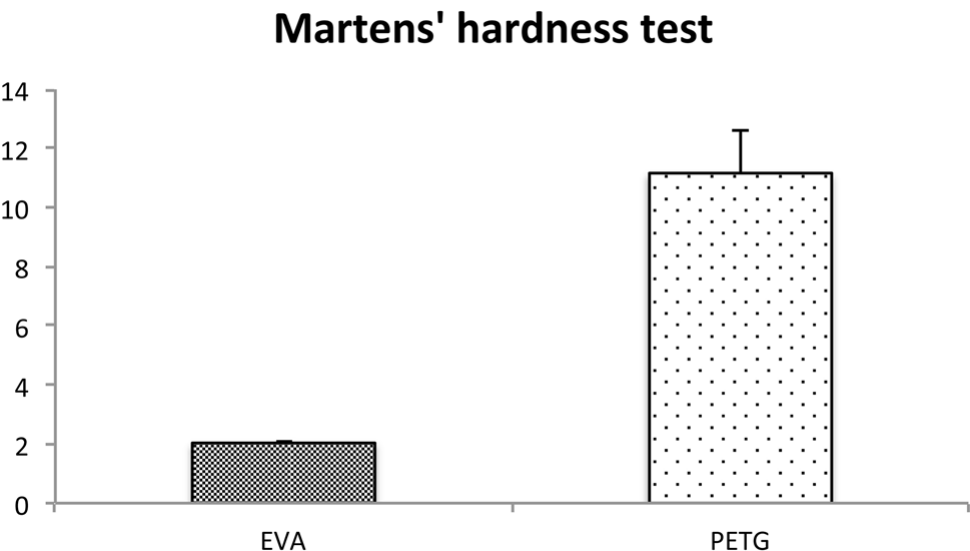
Martens’ hardness test. Martens’ hardness test showed that PTEG (Erkodur^®^) is harder and more rigid than EVA (Erkoflex^®^)

**Fig. 5:**
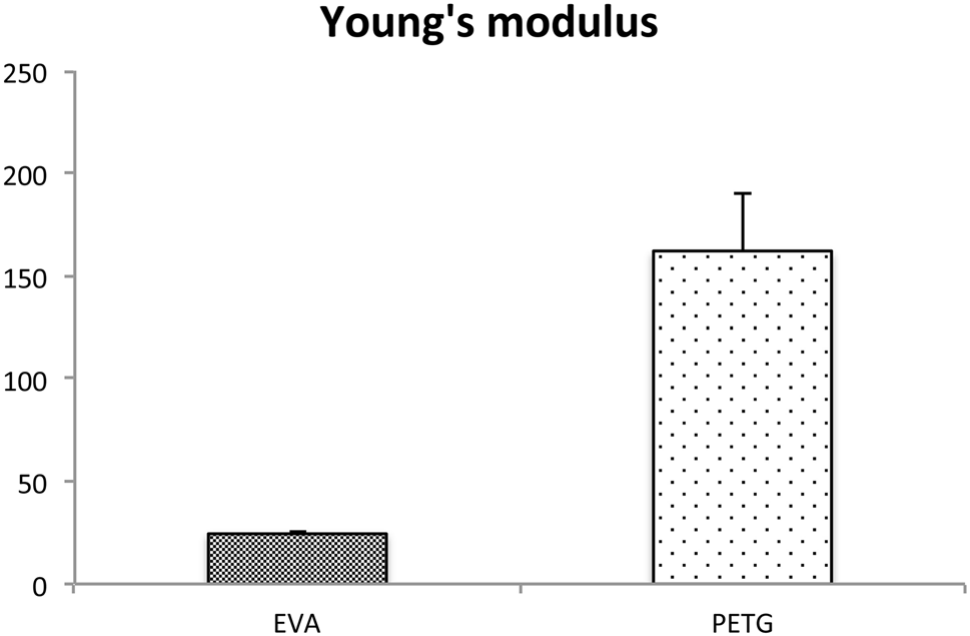
Young’s modulus. Young’s modulus showed that PTEG (Erkodur^®^) is harder and more rigid than EVA (Erkoflex^®^)

To determine the material properties, we measured Martens’ hardness and Young’s modulus. Martens’ hardness was 11.16 ± 1.45 N/mm^2^ for PTEG, and 2.039 ± 0.08 N/mm^2^ for EVA. Martens’ hardness and Young’s modulus for PTEG were significantly higher than for EVA. These findings for Martens’ hardness and Young’s modulus demonstrated that PETG was harder and more rigid than EVA (Figs. 4, 5).

**Fig. 6:**
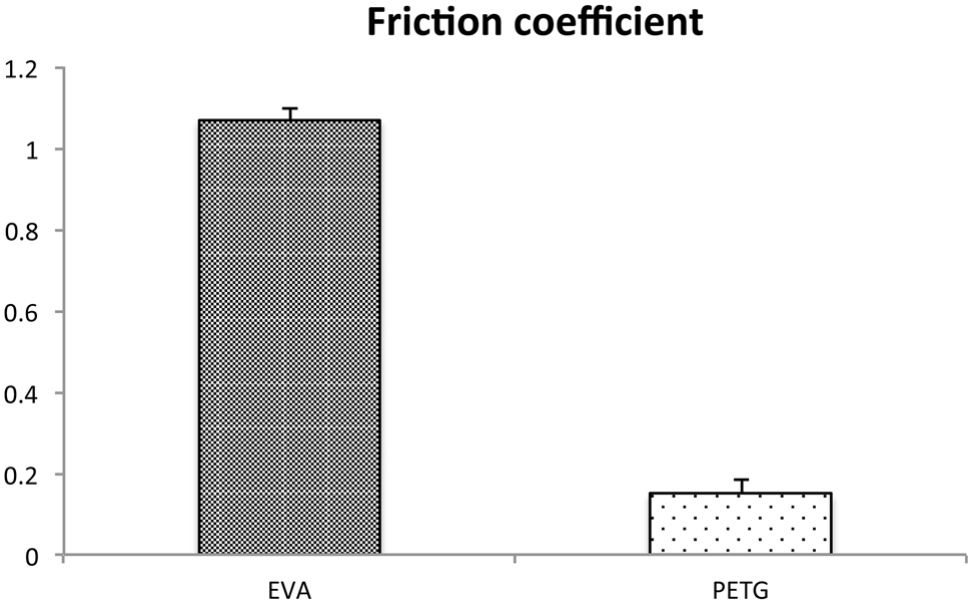
Friction coefficient. Friction coefficient showed that PTEG (Erkodur^®^) is more slippery than EVA (Erkoflex^®^)

To determine the surface characteristics of the two materials, we measured their friction coefficient, which was significantly less at 0.149 ± 0.037 μ for PTEG than for EVA at 1.068 ± 0.027 μ. This result shows that PTEG is more slippery than EVA and thus less likely to concentrate the external force on one point continuously (Fig. 6).

### Load

**Fig. 7:**
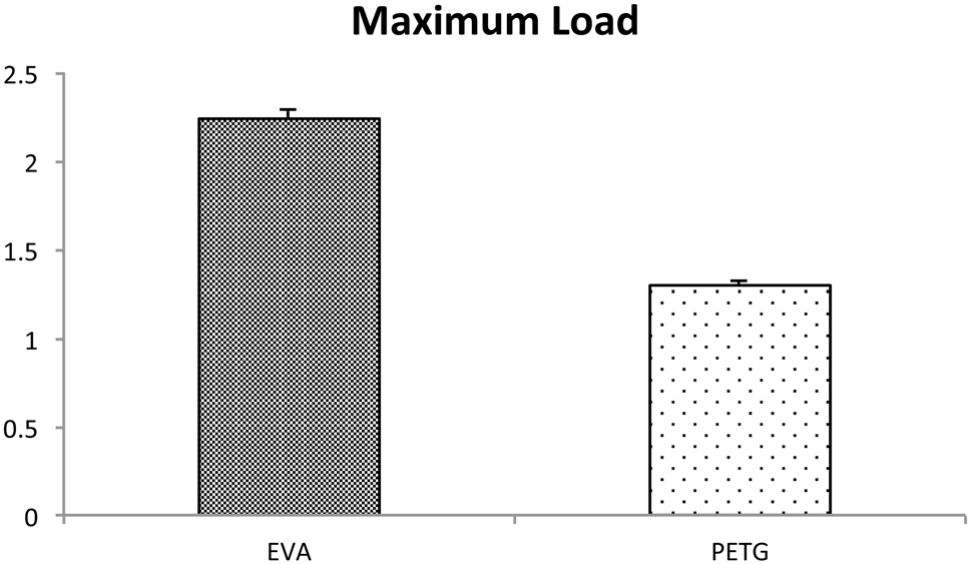
Maximum load. The maximum dynamic load was 1.29 ± 0.03 kgf with the PETG mouthpiece and 2.24 ± 0.05 kgf with the EVA mouthpiece

**Fig. 8:**
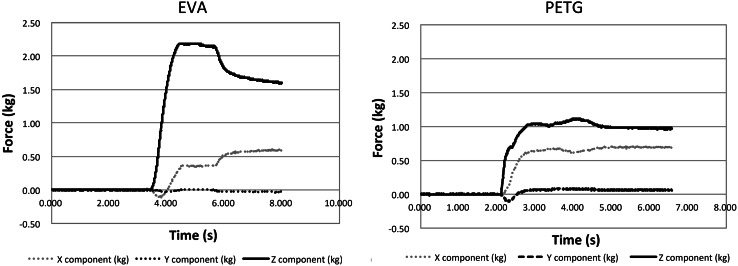
Load. Use of the PETG mouthpiece resulted in less load on the tooth than did use of the EVA mouthpiece

The maximum dynamic load was 1.29 ± 0.03 kgf for the PETG-based mouthpiece and 2.24 ± 0.05 kgf for the EVA mouthpiece (Fig. 7), indicating that with PETG mouthpiece the load against the tooth was significantly less than with the EVA mouthpiece. The direction parallel to the instrument table and anterior–posterior to the mouth model was defined as the *X*-axis and the direction perpendicular to the instrument table and to the *X*-axis as the *Y*-axis. The direction perpendicular to the instrument table was defined as the *Z*-axis. The ratio of component force *Z* to component force *X* was higher for the PETG mouthpiece than the EVA mouthpiece. With the PETG mouthpiece, the direction of the force was more perpendicular to the tooth axis than with the EVA mouthpiece (Fig. 8).

### Strain

The (+) sign for strain means that the strain gauge is elongated in the vertical direction and the teeth are subjected to lateral pressure. The (−) sign for strain means that the strain gauge is shortened in the parallel direction and teeth are subjected to pressure parallel to the axis.

**Fig. 9:**
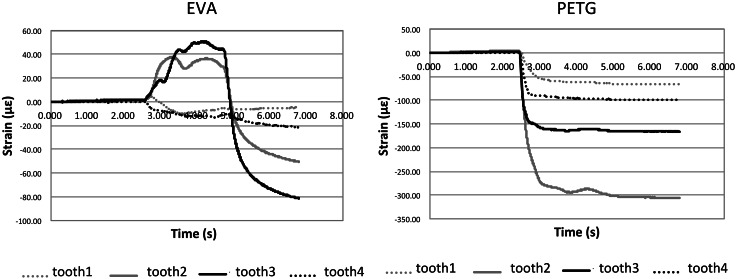
Strain. The load direction was parallel to the tooth axis with the PETG mouthpiece and perpendicular to the tooth axis with the EVA mouthpiece

**Fig. 10:**
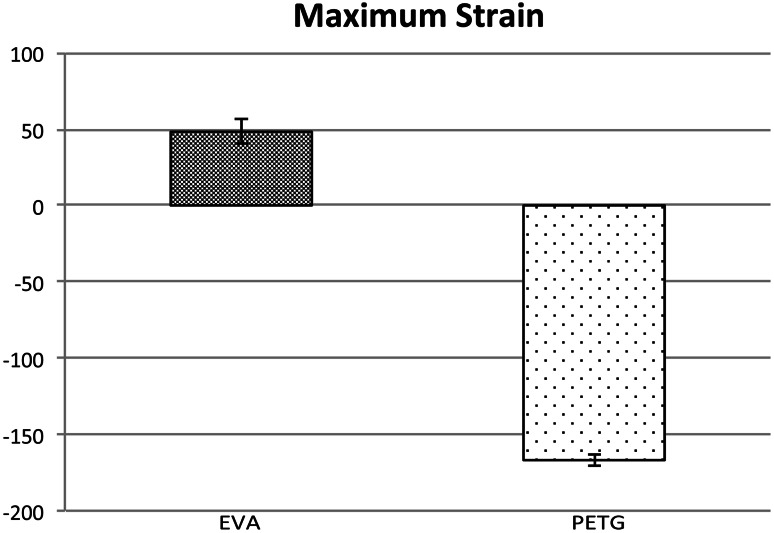
Maximum strain. The maximum strain was 48.24 ± 7.77 με with the PTEG mouthpiece and −166.84 ± 3.94 με with the EVA mouthpiece. The load in the perpendicular direction is likely to result in the loss of teeth, which suggests that the PETG-based mouthpiece offers better protection for the teeth

The strain was positive for the EVA mouthpiece and negative for the PETG mouthpiece. The strain direction was parallel with the tooth axis for the PETG mouthpiece and perpendicular to the tooth axis for the EVA mouthpiece (Fig. 9) with a corresponding maximum strain of 48.24 ± 7.77 and −166.84 ± 3.94 με (Fig. 10). Since a load in the perpendicular direction is likely to result in loss of teeth, this finding suggests that the PETG mouthpiece is more likely to protect the teeth.

## Discussion

Several studies have reported favorable surgical outcomes and preservation of swallowing function with TORS for oropharyngeal cancer, hypopharyngeal cancer, laryngeal cancer, and tumor of the skull base [2–9]. However, tooth injury in 1 % of the patients treated with TORS has been reported [12]. Since the da Vinci surgical system cannot provide haptic feedback, this results in potential risks specific to the transoral use of the da Vinci. The protection of the tissue adjacent to the mouth and pharyngeal area during TORS is essential so that a mouthpiece must be used. Although it is impossible to predict all adverse events that may be associated with new technologies, attempts to assess safety are of paramount importance. However, no assessments of the efficacy of mouthpieces for transoral surgery have been reported. Ours is thus the first study to evaluate mouthpieces for transoral surgery.

In this study, we evaluated the differences between two mouthpieces using a model to determine the degree to which a mouthpiece can prevent risks such as tooth injury resulting from the use of a surgical robot in TORS. The findings of our study suggest that the PETG mouthpiece exerts a smaller load on the tooth load compared with the EVA mouthpiece as a result of a better distribution of the load and rendering the direction of the load parallel to the tooth axis, thus reducing the risk of tooth injury.

The PETG mouthpiece exerted a smaller load on the tooth compared with the EVA mouthpiece. In the dental field, PTEG is used for orthodontic mouthpieces. Several studies have reported how much some materials contribute to the strength of the mouthpiece made for tooth remodeling. The findings of these studies demonstrate that orthodontic forces delivered by thermoplastic appliances depend on the material, thickness, and amount of activation [13–15]. However, no studies have been reported regarding the prevention of tooth damage resulting from the use of a mouthpiece made of PETG and the overall effectiveness of the PTEG mouthpiece. Load measurements performed in our study showed that the PETG mouthpiece reduced the maximum load to the mouth model by half compared with the EVA mouthpiece. Also, material analysis showed that PTEG had lower scores than EVA for Martens’ hardness test and Young’s modulus and was thus more rigid. This finding suggests that a PTEG mouthpiece distributes and thus reduces the load on each tooth. As the reason it was suggested that PETG is more rigid and can, therefore, more effectively distribute the force and reduce the load to the mouth model.

The PETG mouthpiece rendered the load direction parallel to the tooth axis, thus reducing the strain perpendicular to the tooth axis and diminishing the risk of tooth injury. The material analysis performed in this study showed that PETG had a lower friction coefficient compared with EVA, indicating that PTEG is more slippery than EVA. These results show that the PETG mouthpiece distributes the power in the parallel direction and reduces it in the perpendicular direction with the tooth axis. The power perpendicular to the tooth axis involves a much higher risk of loss of teeth than the power parallel to the tooth axis. Our study demonstrated that the PETG mouthpiece reduced the load in parallel with the tooth axis, and shifted the load generated by the external force perpendicular to the tooth axis and thus reduced the risk of loss of teeth.

TORS can damage adjacent tissue for the following reasons. First, the da Vinci surgical system in its present form does not provide haptic feedback and thus poses a potential risk of tissue damage resulting from continuous load to the tissue. Second, what is known as the remote center in da Vinci robotic surgery is the fixed point in the space around which the surgical arm and cannula move and helps in maneuvering instruments inside the surgical site while exerting minimal force on the abdominal or thoracic wall. For TORS, the remote center has to be placed outside the mouth to prevent instruments from hitting each arm. Third, Asian patients have smaller mandibular bones and narrower oral and pharyngeal cavities than their Caucasian counterparts so that surgeons find it difficult to secure sufficient working space. When TORS is used for Asian patients, we, therefore, need thinner and firmer mouthpiece for intra-operative observation and correct placement of the endoscope and instruments. The evaluation of the intraoperative safety of TORS and the utility of various mouthpieces is necessary for prevention of injury.

In the dental field, PTEG is used for the mouthpiece for orthodontics and is widely distributed and easily obtainable in the market place, making the production of mouthpieces using PTEG very easy [13–15]. The material analysis of our study showed that PTEG is hard and rigid, so that with this material a thin and firm mouthpiece can be easily produced which is useful for maximizing the surgical field. The PETG mouthpiece can be inexpensively and quickly made by means of thermoforming. The PETG mouthpiece can be fitted over the patient’s maxillary and mandibular teeth prior to TORS to prevent injury to these teeth.

The PETG mouthpiece reduced the load more than the EVA mouthpiece by distributing the load more. Moreover, the PETG mouthpiece rendered the load direction parallel to the tooth axis and thus reduced the risk of tooth injury. The PTEG mouthpiece is effective for TORS because it reduces tooth injury and allows for a relatively large and wide working space. However, further studies are needed to try and find more appropriate materials for mouthpieces in TORS. Furthermore, we hope to evaluate the load and strain when using the PTEG mouthpiece for real human teeth.
